# Antimicrobial stewardship in residential aged care facilities: need and readiness assessment

**DOI:** 10.1186/1471-2334-14-410

**Published:** 2014-07-23

**Authors:** Ching Jou Lim, Megan Kwong, Rhonda L Stuart, Kirsty L Buising, N Deborah Friedman, Noleen Bennett, Allen C Cheng, Anton Y Peleg, Caroline Marshall, David CM Kong

**Affiliations:** Centre for Medicine Use and Safety, Monash University, 381 Royal Parade, Parkville, VIC 3052 Australia; Department of Medicine, University of Melbourne, Royal Melbourne Hospital, 4th Floor, Clinical Sciences Building, Royal Parade, Parkville, VIC 3050 Australia; Monash Infectious Diseases, Monash Health, Clayton, VIC Australia; Department of Medicine, Monash University, Clayton, VIC Australia; Victorian Infectious Diseases Service, Royal Melbourne Hospital, Parkville, VIC Australia; Department of Infectious Diseases, St Vincent’s Hospital, Fitzroy, VIC Australia; Department of Infectious Diseases, Barwon Health, Geelong, VIC Australia; Victorian Healthcare Associated Infection Surveillance System Co-ordinating Centre, North Melbourne, VIC Australia; Department of Infectious Diseases, Alfred Health, Melbourne, VIC Australia; Department of Epidemiology and Preventive Medicine, Monash University, Melbourne, VIC Australia; Department of Microbiology, Monash University, Clayton, VIC Australia

**Keywords:** Antibiotic prescribing, Antibiotic resistance, Antimicrobial stewardship, Residential care, Qualitative research

## Abstract

**Background:**

Information about the feasibility, barriers and facilitators of antimicrobial stewardship (AMS) in residential aged care facilities (RACFs) has been scant. Exploring the prevailing perceptions and attitudes of key healthcare providers towards antibiotic prescribing behaviour, antibiotic resistance and AMS in the RACF setting is imperative to guide AMS interventions.

**Methods:**

Semi-structured interviews and focus groups were conducted with key RACF healthcare providers until saturation of themes occurred. Participants were recruited using purposive and snowball sampling. The framework approach was applied for data analysis.

**Results:**

A total of 40 nurses, 15 general practitioners (GPs) and 6 pharmacists from 12 RACFs were recruited. Five major themes emerged; perceptions of current antibiotic prescribing behaviour, perceptions of antibiotic resistance, attitude towards and understanding of AMS, perceived barriers to and facilitators of AMS implementation, and feasible AMS interventions. A higher proportion of GPs and pharmacists compared with nurses felt there was over-prescribing of antibiotics in the RACF setting. Antibiotic resistance was generally perceived as an issue for infection control rather than impacting clinical decisions. All key stakeholders were supportive of AMS implementation in RACFs; however, they recognized barriers related to workload and logistical issues. A range of practical AMS interventions were identified, with nursing-based education, aged-care specific antibiotic guidelines and regular antibiotic surveillance deemed most useful and feasible.

**Conclusions:**

Areas of antibiotic over-prescribing have been identified from different healthcare providers’ perspectives. However, concern about the clinical impact of antibiotic resistance was generally lacking. Importantly, information gathered about feasibility, barriers and facilitators of various AMS interventions will provide important insights to guide development of AMS programs in the RACF setting.

**Electronic supplementary material:**

The online version of this article (doi:10.1186/1471-2334-14-410) contains supplementary material, which is available to authorized users.

## Background

There is increasing evidence that the elderly population in residential aged care facilities (RACFs) serves as an important reservoir for multidrug-resistant (MDR) organisms [1, 2], with high antibiotic use causing selective pressure and encouraging the emergence of various MDR organisms [3, 4]. As antibiotic resistance in bacteria increases and the development pipeline of new antibiotics declines, judicious use of antibiotics through antimicrobial stewardship (AMS) programs has become critical across all parts of the healthcare system, including the RACF setting. Formal AMS programs have been increasingly established in the acute-care hospital setting, but remain relatively uncommon in RACFs [5].

Essentially, the need for AMS and the potential areas for AMS interventions are reliant upon existing antibiotic use and resistance patterns. For example, a study in Australian RACFs has shown less frequent use of broad-spectrum antibiotics such as fluoroquinolones [6] compared to long-term care facilities in the United States (US), where fluoroquinolone prescribing is widespread [7, 8]. Likewise, the magnitude of antibiotic resistance has been reported to vary across RACFs in different geographical areas [9]. Not surprisingly, surveys involving Nebraska and Irish long-term care facilities have reported very distinctive AMS practices, presumably because AMS interventions are tailored according to the needs and resources of individual institutions in different geographical areas [10, 11].

International guidelines for infection control and prevention have strongly recommended initiation of AMS programs in the RACF setting [12]; however, practical models for AMS in this setting remain poorly delineated. Adopting hospital-based AMS programs in the RACF setting may be unrealistic due to differences in organizational resources and antibiotic prescribing patterns between these two healthcare settings. Recent studies from the US reported that AMS interventions involving multidisciplinary teams with regular audits are effective in reducing inappropriate antibiotic prescribing in the long-term care setting; however, these studies were mainly single centre and their sustainability remains in question [13, 14]. To date, information about the feasibility, barriers and facilitators of AMS programs in RACFs has been scant. The perceived need and readiness for AMS interventions in the RACF setting can be explored via qualitative research that allows in-depth investigation into the social and environmental determinants of antibiotic prescribing practices [15]; however, such work had previously not been undertaken. In preparation for developing practical and sustainable AMS models in this setting, the current study explored the attitudes and perceptions of key healthcare providers towards AMS interventions in Australian RACFs.

## Methods

### Study population and setting

This is part of a larger study exploring key healthcare providers’ views about improving antibiotic use in the RACF setting. The study targeted primarily high-level care RACFs (i.e. nursing homes) affiliated with four major public healthcare service networks within metropolitan and regional Victoria, Australia. These residential care facilities deliver 24-hour nursing care to elderly residents requiring significant assistance in their activities of daily living. There was no institutional policy for antibiotic prescribing in any of the RACFs; however, intravenous antibiotic therapy when required is normally delivered via specialized support from hospitals.

Three major groups of healthcare providers servicing the participating RACFs were invited to participate, namely general practitioners (GPs), nurses and pharmacists. At these RACFs, the medical care is provided by off-site GPs (equivalent to family physicians in the US) from different practices, who visit residents periodically or upon request. There are significant roles for nursing staff in daily care of residents, including ringing GPs to request medical assessment. Prescription medicines, including antibiotics, require an order from the GPs and are supplied by external community pharmacies. Medication review for individual residents [known as Residential Medication Management Review (RMMR)] is normally performed on an annual basis by consultant pharmacists, however this does not involve audit of short-term antibiotic use.

### Participant recruitment and data collection

Institutional ethics approvals from the human research ethics committees of all participating healthcare service networks and Monash University were obtained prior to participant recruitment. A combination of purposive and snowball sampling strategies were used for recruitment of different healthcare providers [16]. The aforementioned healthcare professionals with routine involvement in the antibiotic prescribing process were intentionally approached (i.e. purposive sampling), and some other participants were also recruited through recommendation by initial informants (i.e. snowball sampling). Informed consent was obtained from individual participants, and participation was voluntary.

Nursing staff in different clinical positions [senior executive nurses, nurse unit managers (NUMs), registered nurses (RNs)] were involved in either one-on-one interviews or focus groups. All NUMs and RNs were involved in daily care of residents, whilst the executive nursing staff were responsible at the policy-making level for quality improvement of resident care. Face-to-face or phone interviews were conducted with the GPs and pharmacists, depending on their preference. We used several triangulation strategies; we sought information from different stakeholders’ perspectives (i.e. data triangulation), performed onsite observation on the organizational workflow and documentations related to antibiotic prescribing (i.e. methodological triangulation), and explored views of participants from RACFs in different locations (i.e. environmental triangulation).

All interviews were conducted using a semi-structured interview guide, which was tailored to different healthcare providers (Additional files 1, 2 and 3). The interview guide was divided into three main domains (antibiotic prescribing, antibiotic resistance and AMS) whilst allowing flexibility to pursue particular issues by more in-depth discussion as they emerged from the interviews. All discussions were moderated by one or two interviewers (CJL and MK). Recruitment of key stakeholders from the four healthcare networks continued until data saturation (i.e. when no new relevant themes emerged). Participant recruitment and interviews were conducted between January and July 2013. All interviews were audio-recorded and transcribed verbatim by an independent, professional transcribing service.

### Data analysis

Data were analyzed for emergent themes using the framework approach, as described elsewhere [17, 18]. This approach involved five stages: (i) familiarization with the data collected by detailing the interview recording and transcripts; (ii) identifying key issues and themes that construct a thematic framework; (iii) indexing (coding the data) into themes; (iv) charting by rearranging indexed data according to the thematic framework; and (v) mapping and interpreting the data. Data management of interview transcripts and recording was facilitated using Nvivo® 9.0 (QSR, Melbourne). All transcripts were verified against audio recordings by CJL and MK. Data analyses were carried out independently by the two researchers (CJL and MK) for cross-validation purpose, with peer-debriefing at regular intervals. Themes and codes were discussed at regular meetings involving all co-authors, where discrepancies were resolved and themes were finalized.

## Results

Twelve high-level care RACFs within the four major healthcare networks participated. From these RACFs, 40 nursing staff [four executive nurses, 15 nurse unit managers (NUMs), and 21 registered nurses (RNs)], 15 GPs and six pharmacists consented to participate in the study. The majority of participants were interviewed individually, with 15 RNs participating in three focus groups (range 4–6 RNs per focus group). All except four interviews were conducted face-to-face with participants. The demographic characteristics of the participants are described in Table 1. Five major themes that illustrate the prevailing perceptions and attitudes towards the need and readiness for AMS program in this setting emerged:

**Table 1:** **Demographic characteristics of participants**

Characteristics	Nurses, n = 40	GPs, n = 15	Pharmacists, n = 6
**Age, n (%)**			
**≤40 years**	11 (28)	0	2 (33)
**>40 years**	29 (73)	15 (100)	4 (67)
**Female, n (%)**	37 (93)	7 (47)	4 (67)
**Mean years of work experience in RACF, (range)**	13 (0.75 - 43)	22 (4–40)	8.5 (4–12)
**Locations, n (%)**			
**Metropolitan**	32 (80)	10 (67)	5 (83)
**Regional**	8 (20)	5 (33)	1(17)

NOTE. GPs = general practitioners; RACF = residential aged care facility.

### Perceptions of current antibiotic prescribing behaviour

There were mixed views about existing antibiotic prescribing behavior (Table 2). Several nurses and pharmacists believed that current antibiotic use in RACFs was not excessive; most indicated that perceived patient frailty or behavioral changes often precluded the potential strategy of withholding antibiotic treatment and observing for development of further clinical signs. Likewise, a few GPs felt that empiric prescribing of broad-spectrum antibiotics was fairly reasonable for the elderly population in RACFs (Table 2, Q1-Q3). In contrast, twelve of 15 GPs perceived that there was over-prescribing of antibiotics, with many admitting to prescribing antibiotics “just in-case” in light of the potential risk for patient deterioration if treatment was not initiated. A number of GPs indicated that pressure from nursing staff and family members was an important reason leading to unnecessary antibiotic prescribing (Table 2, Q4- Q5). Relatively fewer nurse participants were concerned about excessive antibiotic use among RACF residents. Those nurses who were concerned felt that there was liberal prescribing of antibiotics for futile reasons including viral illness and asymptomatic bacteriuria. Some were concerned about frequent empiric antibiotic prescribing without microbiological investigations to confirm causative organisms (Table 2, Q6-Q7). The main concern raised by participating pharmacists was about prolonged durations of antibiotic treatment (Table 2, Q8). They indicated that antibiotics were generally prescribed for an average of 7–10 days; however, it was not uncommon for antibiotics to be administered for longer periods when doctors had not documented a planned cessation date for treatment of acute infections, or where antibiotics were utilized for long-term prophylaxis against infection.

**Table 2:** **Perceptions of current antibiotic prescribing behaviour and antibiotic resistance in the residential aged care facility setting**

Themes	Positive views	Negative views
**Perceptions of current antibiotic prescribing behaviour**	**Q1.** “It [Antibiotic use] is probably about right, I’ve sort of never had an issue where I’ve thought ‘oh no they should have been on antibiotics or yeah they should not be’, and as I said a lot of our [residents], you’ve got to weigh up the [change of] behaviours and the risks to everyone.” **(NUM 13, 30 yr)**	**Q4.** “I guess the other thing is that we know particularly in the elderly, it’s better to get in early and treat. So if you think should I/shouldn’t I, then treat, you know, rather than wait until they get crook. And if it’s a bit of over treatment so be it.” **(GP 7, 30 yr)**
	**Q2.** “It’s usually pretty reasonable. Because bearing in mind that where it may be a viral infection initially, a lot of these people are frail, non-ambulant and will go on to a secondary bacterial infection.” **(Consultant pharmacist 5, 12 yr)**	**Q5.** “Probably an over use, because we get pressured by nursing staff mostly to prescribe, they're very reluctant to let anyone with a cold be untreated.” **(GP 1, 32 yr)**
	**Q3.** “We don’t have the luxury of if this antibiotic doesn’t work we’ll try something else, often they [RACF residents] can go down so fast…You don’t have that second chance, so you’ve really got to hit them [with broader spectrum antibiotics].” **(GP 13, 30 yr)**	**Q6.** “It’s increased for everything; someone sneezes they get antibiotics, somebody’s urine smells or you know it lights up a stick, they get antibiotics.” **(NUM14, 12 yr)**
		**Q7.** “… some GPs continually use the same antibiotics, you know people are put on the same antibiotics for maybe 2 months….they don’t think to go and check to see if they're resistant or whatever any more.” **(NUM 7, 9 yr)**
		**Q8.** “Well, there might be an infection, they just go in there and write up a broad-spectrum [antibiotic] and have it for 2 weeks regardless of what the infection is. They're not too much adhering to the [Australian national] antibiotic guidelines.” **(Community pharmacist 1, 7 yr)**
**Perceptions of antibiotic resistance**	**Q9.** “I wouldn’t say there's been any change [of antibiotic resistance trend] in all the time I’ve practiced medicine…Probably I don’t do that much pathological testing, but I don’t see it as having been an issue.” **(GP 12, 5 yr)**	**Q11.** “I would think so, yes, I think you get more of the really unusual urine reports and unusual bacteria or ones with multi resistance, yeah for sure…I guess anywhere where there's a lot of antibiotic use there's going to be a lot of resistance generated.” **(GP 1, 32 yr)**
	**Q10.** “In the nursing home setting it’s not a major issue…I’d have to look up their [residents’] notes to see [if they have MDR organism infection or colonisation]…It’s not a big issue for us because we are not a hospital setting, so we wouldn’t isolate someone for example who’s VRE [vancomycin-resistant enterococci] positive.” **(NUM 1, 13 yr)**	**Q12.** “From a wound swab point of view, you're more likely to get a resistant bug in an aged care facility than you are in the community…” **(GP 2, 20 yr)**
		**Q13.** “It used to be a really big deal … if someone had MRSA, ‘oh my God’ you know it was the yellow bags and everything came out and we isolated them and we would scrub. Now I mean we wouldn’t even blink if [we see these MDR organisms]. And I don’t think the younger staff realise too the implications of someone having MRSA. But now it's nothing, no one really cares.” **(NUM 10, 23 yr)**

NOTE. Q = quotes extracted from interview transcripts; NUM = nurse unit managers; GP = general practitioner; yr = years (of work experience in RACF).

### Perceptions of antibiotic resistance in RACFs

There were also mixed perceptions about antibiotic resistance (Table 2). Several GPs claimed they had not encountered many MDR organisms within their clinical practice; however, they admitted that cultures of relevant clinical samples were seldom requested. Similarly, the majority of nursing staff did not see this as an important issue, as they believed it was encountered less frequently than in the hospital setting, and often did not change management of residents within the RACFs (Table 2, Q9-Q10). In comparison, about half of the GPs believed that antibiotic resistance was an emerging issue in RACFs, reporting that MDR organisms were often seen in residents with recurrent urinary tract infections (especially those with indwelling urinary catheters), long-term antibiotic prophylaxis and chronic wound colonization (Table 2, Q11-Q12). However, only a minority of GPs were concerned that antibiotic resistance would affect their choice of empiric antibiotics. Whereas from the nurses’ perspective, only a small proportion were worried about the increasing rates of MDR organisms; their main concerns were about low staff awareness and inadequacy of existing infection control efforts in preventing MDR organism transmission as opposed to clinical impact on residents (Table 2, Q13).

### Attitude towards and understanding of AMS

The majority of participating nursing staff were unaware of the concept of AMS. In comparison, more GPs and pharmacists were aware of AMS, although they generally felt that AMS was relatively new in the RACF setting. AMS refers to integrated activities that help to optimize antibiotic therapy, ensuring the best clinical outcomes whilst minimizing the risk for the emergence of antimicrobial resistance. When this concept of AMS was explained, in general, all key stakeholders were supportive of AMS programs in RACFs. Amongst GPs, the main value of AMS was thought to be in promoting evidence-based practices for antibiotic prescribing in this setting (Table 3, Q1). All executive nursing staff felt that AMS was applicable, and welcomed future intervention as part of quality improvement strategies in their RACFs (Table 3, Q2). Likewise, the NUMs and RNs felt that AMS interventions would be helpful as an additional source of educational support for nurses, given their relative lack of knowledge regarding antibiotic use (Table 3, Q3-Q4). The pharmacists were also supportive of AMS interventions, particularly to achieve more uniformity in antibiotic prescribing and consistency with adopting guidelines (Table 3, Q5-Q6).

**Table 3:** **Perceptions of implementation of antimicrobial stewardship from different healthcare providers’ perspectives**

Themes	Representative quotes from different healthcare providers’ perspectives		
	GPs	Nurses	Pharmacists
**Attitude towards AMS interventions**	**Q1.** “[We know] that the resistant organisms are becoming more and more of a bigger problem. And our aged care group have got an increased incidence of side effects from the antibiotics, and it's not uncommon for us to end up with someone with diarrhoea for example plus or minus *Clostridium difficile* colitis. So you know, thrush is a big problem in this sector as well through antimicrobials. So there are lots of reasons why we should try to adhere to the best practice.” **(GP 14, 27 yr)**	**Q2.** “Yes, I think it’s certainly applicable and I think it’s been identified that we need to improve our practice. We would be prepared to do some sort of a follow-up project…” **(Executive nurse 4, 12 yr)**	**Q5.** “It [Antimicrobial stewardship] is needed…In most cases, the registered nurses, or the enrolled nurses, cannot make a decision about whether an antibiotic is needed or not.” **(Consultant pharmacist 5, 12 yr)**
		**Q3.** “It would be good, so only right antibiotics being used and only [prescribed to] the right resident. Only residents really need to be given [antibiotics] rather than things like behaviour change is [presumed to be] UTI and give antibiotics.” **(NUM 4, 20 yr)**	**Q6** ***.*** “I think it’s fantastic because the next step, aged care, is like a hospital or if you wanted to say that, where they are in a controlled environment where their medications are, you know, freely given but they are [supposed to be] given to them accordingly.” **(Community Pharmacist 4, 4 yr)**
		**Q4.** “To be honest many of us don’t think we are overusing antibiotics in cases where it might be over using antibiotics…So if you want to change it you should be teaching and educating us.” **(RN 7, 2 yr)**	
**Perceived barriers and facilitators**	**Q7.** “I think that there is always the question of … doctor autonomy, you know, that doctors like to be their own boss, don’t like to be dictated to.” **(GP 8, 20 yr)**	**Q9.** “In residential aged care there's a burden of workload, a burden of lots of things, documentation etc., so there's always a risk of ‘oh this is just another thing’.” **(Executive nurse 2, 4.5 yr)**	**Q13.** “So obviously in hospital setting it’s a bit more intimate because the doctors and the pharmacists are liaising with one another quite frequently as opposed to a community pharmacist and the aged care doctors.” **(Community pharmacist 4, 4 yr)**
	**Q8.** “There's too many people [GPs] dabbling…So I think if we got more people doing [full-time] aged care, or having more residents in the one facility, a lot of these problems within residential care will diminish, because you’ll get more consistent treatments.” **(GP 14, 27 yr)**	**Q10.** “So GPs [are] in their own practices, they have their own perhaps ways of doing things… that may present some barriers to introducing something like that…” **(NUM 1, 13 yr)**	**Q14.** “I’d be more than happy to use resources that you provide on you know best practice, guidelines, whatever, and then educate [nursing] staff. I mean I would have no problem in that and yes there is a role.” **(Consultant pharmacist 6, 12 yr)**
		**Q11.** “What we’ve found in the past with other similar things is to have a champion, where we have a couple of people trained up who drive the program, and bring their peers on board if you like.” **(Executive nurse 3, 10 yr)**	
		**Q12.** “Obviously all the parties need to have that education…we need to get it across to everyone, so I think nursing staff are the best people to do that, to go up and to go down at the same time._”_ **(NUM 8, 12 yr)**	

Note. Q = quotes extracted from interview transcripts; GP = general practitioner; NUM = nurse unit managers; RN = registered nurse.

### Perceived barriers to and facilitators of AMS interventions

A number of perceived barriers and facilitators in relation to implementation of AMS interventions were raised. From the GPs’ perspective, several raised concerns about the potential for doctor autonomy to hinder acceptance of institutional policies and guidelines. Heterogeneity in prescribing practices amongst GPs from different practices pose another barrier, thus having fewer GPs with greater patient loads working in each facility would promote more consistent prescribing practices (Table 3, Q7-Q8). Nursing staff had mixed attitudes. The executive nursing staff anticipated that AMS interventions might not be easily accepted by nursing staff in view of their high workload. Conversely, most of the NUMs did not foresee major resistance from nursing staff given their past experiences with other quality improvement initiatives, instead, having more concern about GP acceptance of any AMS interventions (Table 3, Q9-Q10). It emerged that nursing staff would need to play an essential role in delivering any future AMS intervention, with facilitation of AMS programs being driven by on-site staff such as NUMs or clinical nurse coordinators. The nursing staff would be in a good position to disseminate relevant information to family members and GPs (Table 3, Q11-Q12). Both community and consultant pharmacists perceived several logistical barriers to providing additional clinical support, in particular, their lack of on-site availability, inadequate access to patients’ clinical information, and limited communication with GPs. All consultant pharmacists were willing to undertake additional clinical roles; however, they acknowledged that current funding resources offered little prospect for this to occur (Table 4, Q13-Q14).

### Feasible AMS interventions

Three major areas of AMS interventions emerged as potentially useful and practical in the RACF setting (Table 4). The most commonly suggested intervention was ongoing education to nurses, GPs and family members of residents, with the objective of promoting awareness of judicious antibiotics use amongst those working in the RACFs (Table 4, Q1-Q3). Various suggestions for how this could be achieved were identified, including in-service training (for nurses), web-based education, provision of educational material in poster or brochure form, and invited speaker sessions. Another area identified was the emphasis on evidence-based practice around aged-care specific management. Importantly, more than half of the GPs indicated a need for aged care-specific antibiotic guidelines, as current guidelines were thought to be lacking evidence and recommendations specific to the aged care population. The nursing staff perceived a need for consistent, RACF-based guidance and support on the matter of infection management; for example, a clinical protocol guiding the management of symptomatic versus asymptomatic bacteriuria was frequently recommended (Table 4, Q4-Q6). The third main potential area for future AMS identified was surveillance and auditing of antibiotic use. It was believed that monitoring of antibiotic use with regular feedback to GPs would be helpful to guide and target reduction of specific antibiotic use, and consultant pharmacists were deemed most suitable to perform such activity (Table 4, Q7-Q9). In contrast to more proactive hospital-based AMS interventions (such as pre-authorization of broad-spectrum antibiotics or review by infectious diseases teams), passive surveillance of antibiotic use was thought to be sufficient and more practical in this setting. Other suggestions included additional staff resources via introduction of the nurse practitioner model, and re-institution of aged care interest groups to further support and educate GPs.

**Table 4:** **Feasible antimicrobial stewardship interventions deemed useful by three key stakeholders**

AMS interventions deemed most useful	Representative quotes from different healthcare providers’ perspectives		
	GPs	Nurses	Pharmacists
**Education**	**Q1.** “They (GPs) are very much used to their general practice, but the treatment of geriatric case load is different…well obviously educating the GPs is really important.” **(GP 14, 27 yr)**	**Q2.** “We all talk about the 5 rights of medication, and I’m sure there are 5 rights about antibiotic use. So you know just reminding people, are we creating a superbug, are we giving antibiotics when they're not required.” **(NUM 8, 12 yr)**	**Q3.** “But if we could educate the nurses who are there every day, you know at the coalface, when they could see the doctor’s keeping prescribing [antibiotics], they could just bring it up with the GP…” **(Consultant pharmacist 2, 12 yr)**
**Policy and guidelines**	**Q4.** “… the [antibiotic] therapeutic guidelines might say okay for a pharyngitis you might use this [antibiotic], in a penicillin allergic patient you’ll use this or for a community acquired pneumonia you’ll use this, and for a hospital acquired pneumonia this is what we recommend…I think it’d be useful if they [guidelines] were there, to sort of say well you know based on trials we’ve found that nursing home patients need two courses or they don’t need any more than the regular.” **(GP 3, 12 yr)**	**Q5.** “Some sorts of protocol like when, say for UTI, when to start antibiotics, what stage of infection, signs and symptoms you have to really go on antibiotics. For respiratory infections when the person has to go on antibiotics, and when we see what kind of signs and symptoms definitely should be on antibiotics or something like that, would be helpful I think.” **(RN 14, 9 months)**	**Q6.** “So if you have got universal policies on infection control and antibiotic uses, you have got a bit of a chance [to facilitate consistent antibiotic prescribing practices].” **(Consultant pharmacist 5, 12 yr)**
**Surveillance/auditing of antibiotic use**	**Q7.** “Because well I always prescribe Abbocillin® for tonsillitis but it’s amazing how many people prescribe Amoxil® or Ceclor® or Keflex® or something like that, you know. And yeah so obviously that kind of auditing is a good idea.” **(GP 8, 20 yr)**	**Q8.** “I think it's helpful to do [antibiotic surveillance], because we do know that sometimes people are left on medications for too long, and unless someone’s got that awareness of it to say ‘I don’t think this might be working’.” **(Executive nurse 3, 10 yr)**	**Q9.** “Just getting a general idea and another set of eyes on the actual outcome of the patient would definitely help with the prescribing habits as well… I believe that obviously RMMR [consultant] pharmacists they can come in because they get access more to clinical notes than what a community pharmacist would do.” **(Community pharmacist 4, 4 yr)**

Note. Q = quotes extracted from interview transcripts; GP = general practitioner; NUM = nurse unit managers; RN = registered nurse; yr = years (of work experience in RACF); RMMR = Residential Medication Management Review.

## Discussion

To our knowledge, this is the first study exploring the perceptions and attitudes of a range of healthcare providers towards antibiotic prescribing behaviour, antibiotic resistance, and AMS implementation in the RACF setting. A greater proportion of GPs and pharmacists than nursing staff felt that there was over-prescribing of antibiotics, suggesting a lack of awareness amongst nurses about potential antibiotic misuse among this population. Antibiotic resistance and the emergence of MDR organisms were perceived as more of a concern from the infection control perspective as opposed to impacting empiric antibiotic selections. Additionally, this study has highlighted the prevailing attitudes amongst key healthcare providers that AMS interventions were needed and deemed useful in the RACF setting. A number of perceived barriers to AMS programs were identified, in particular, nursing staff workload and the logistical issues of off-site GPs and pharmacists. A range of potential AMS interventions have been suggested to provide insights into feasible AMS model for the RACF setting.

Published data describing the key stakeholders’ views of antibiotic prescribing and antibiotic resistance have mainly focused on the general practice setting, with limited information from the RACF setting [18, 19]. A study by Walker *et al.* has explored the views of the physicians and nurses about antibiotic prescribing in Canadian RACFs but it focused specifically on treatment for asymptomatic bacteriuria, reporting that education about asymptomatic bacteriuria was viewed as an important priority by both physicians and nurses [20]. In addition to concern about antibiotic treatment for asymptomatic bacteriuria, our study also identified a need for education to target other infective issues, including the widespread prescribing for viral upper respiratory tract infections, and repeated or prolonged antibiotic use without microbiological investigation. The current study found that the overuse of antibiotics in the Australian RACFs context was thought to be mainly related to widespread empiric prescribing or unnecessary antibiotic treatment, as opposed to the over-prescribing of broad-spectrum antibiotics such as intravenous antibiotics and oral fluoroquinolones that was more frequently reported in the US studies [7, 8].

Previous studies have reported that increasing the awareness about antibiotic resistance would potentially influence GPs’ decisions in selecting antibiotics, underlining the importance of knowledge about MDR organisms in assisting clinical decisions [18, 21, 22]. Furthermore, education that promotes awareness about antibiotic resistance is likely to encourage more microbiological testing to identify causative organisms before initiating antibiotic treatment. Several international guidelines suggest that provision of antibiograms by local microbiology laboratories as fundamental requirement for an AMS program in the RACF setting [12, 23]. However, the need for antibiograms to guide empiric antibiotic therapy has not been suggested as a useful or practical AMS initiative by any healthcare provider that participated in this study. The feasibility of this strategy may be hindered by limited microbiological investigations and involvement of multiple external pathology laboratories.

AMS interventions in the RACF setting have been few despite mounting evidence of inappropriate antibiotic prescribing among this elderly population [9, 24]. Existing guidelines about the implementation of AMS have primarily been limited to the acute-care hospital setting [25]. An intensive form of AMS intervention with involvement of infectious diseases physicians or clinical pharmacists has been adopted in Veterans Affairs long-term care facilities in the US [14]; however, most key stakeholders in the present study indicated that such AMS interventions are not practical or necessary in the Australian RACF setting. From the current work, an approach to AMS tailored to the needs of key healthcare providers in RACFs is proposed (Table 5). Multifaceted interventions are likely to be most effective; however, such interventions should be tailored to the resources and expertise in individual RACFs. Overall, the important role of nursing staff in the day-to-day practice of AMS in RACFs cannot be under-estimated, and could function effectively if supplemented by education, infection management algorithms and training in the use of antibiotic utilization surveillance.

**Table 5:** **Potential areas of focus for antimicrobial stewardship interventions as proposed by study participants**

AMS interventions	Potential areas of focus
Nursing staff education and training	-Reinforcement of issues about antibiotic use, antibiotic resistance and benefits of antimicrobial stewardship
	-Education sessions as part of in-service training, via online training modules, awareness campaigns, etc.
Aged care-specific infection management algorithms for nursing staff	-Flow charts to guide appropriate initial testing of residents, as well as guidance with regard to conditions that may be observed versus those requiring immediate contacting of prescribers
	-Targeted management of common infections, in particular, urinary and respiratory tract infections
Aged care-specific antibiotic treatment guidelines	-Development of evidence-based aged care antibiotic treatment guidelines (with recommendations about appropriate dosages and duration of therapy)
	-Education to prescribers through online updates or distribution of newsletters, highlighting evidence-based prescribing practices
Regular surveillance of antibiotic use by consultant pharmacists	-Passive surveillance and audit of antibiotic use, with regular feedback to the prescribers
	-Documentation of individual residents’ prior antibiotic exposure over time, supplemented by antibiotic susceptibility results to guide prescribing decisions
Improved communication about decisions related to antibiotic prescribing	-Early discussion with residents and/or families about antibiotic use during acute events and terminal illness as part of advanced care planning
	-Proper handover from locum doctors to regular GPs regarding antibiotics prescribed after hours for further review
	-Faxing of treatment plans for phone ordering of antibiotics via antibiotic ordering form with clear indications for treatment and planned duration of treatment

This study has predominantly involved high-level care hospital-affiliated RACFs. There may be differences in antibiotic prescribing behaviour in comparison to low-level care or private RACFs, and thus this study could be replicated more widely to other RACF setting for further exploration. Given the differences in long-term healthcare delivery models between different countries [26], the findings and suggestions in the current study may not be generalizable outside the Australian setting. Nevertheless, the current findings are likely to be of interest to many, especially those who are closely affiliated with RACFs. Participation in this study was voluntary, thus the expression of personal perceptions may be skewed towards those who are more involved with or concerned about AMS. In order to minimize the potential bias, we have explored both the positive and negative views from a range of key stakeholders, and continued the recruitment and data analysis until we achieved data saturation amongst all stakeholder groups.

## Conclusions

In summary, AMS interventions have been deemed applicable and useful by the key stakeholder groups involved in the provision of healthcare in RACFs. Potential barriers and facilitators for AMS interventions have been highlighted, providing important information to guide allocation of future AMS resources. More importantly, the major areas of AMS deemed most needed and practical in the RACF setting have been identified to guide the development of a feasible AMS model.

## Authors’ information

Caroline Marshall and David CM Kong are senior authors.

## Electronic supplementary material

Additional file 1: Semi-structured interview guide for general practitioners.(PDF 216 KB)

Additional file 2: Semi-structured interview guide for nursing staff.(PDF 209 KB)

Additional file 3: Semi-structured interview guide for pharmacists.(PDF 155 KB)

## Abbreviations

AMS: Antimicrobial stewardship
RACFs: Residential aged care facilities
GP: General practitioners
MDR: Multidrug-resistant
US: United States
RMMR: Residential medication management review
NUM: Nurse unit manager
RN: Registered nurse.
