# Supplementary material for: Antimicrobial stewardship in residential aged care facilities: need and readiness assessment
Source: BMC Infect Dis. 2014 Jul 23;14:410. doi: 10.1186/1471-2334-14-410 (PMC4117949; doi:10.1186/1471-2334-14-410)
Supplement: Supplementary file 1 — Additional file 1: Semi-structured interview guide for general practitioners.(PDF 216 KB) [file 12879_2014_3703_MOESM1_ESM.pdf]

INTERVIEW GUIDE - GENERAL PRACTITIONER

1. PRESCRIBING WORKFLOW/ ORGANISATIONAL CULTURE

- GP workload:
  - How many patients do you have in aged care?
  - On average, much time in a week would you spend attending to residents at a RACF (eg. regular rounds, urgent calls)?

- Assessment and follow-up of patient:
  - How do you go about making the decision to prescribed antibiotics for residents in aged care setting and monitor patient's progress?
  - When you are contacted about an unwell patient, how do you carry out further assessment? (eg. over phone vs in person, how long until assessing in person)
  - What if you can't attend urgently eg. after-hours? (use locum, ask facility to contact HITH/InReach/MATS, or send to ED?)
  - Over the phone, how do you decide if an infection is likely? (eg. ask observations, urinalysis, infective symptoms eg cough, SOB, diarrhoea, coryza, rigors etc)
    - How do you feel about the nurse assessments? (try to prompt issues about nurses' knowledge in recognising s/s of infections, timeliness of reporting)
  - If you think an infection is likely, do you always order investigations? (if yes, which ones/which infections)
  - What factors make you more likely to prescribe an antibiotic vs watch and wait for someone in aged care facility?
    - Signs/symptoms of patient, severity of illness, patient with difficult behaviour, patients with advanced directives
    - How do the opinions/attitudes of nursing staff, family or relatives influence your antibiotic prescribing?
  - How do you follow-up the progress of a resident with an infection, and within what timeframe? (eg. communicating with staff at RACF, review in-person)
    - Would you always review patients in-person? (eg. after seen by locum after hours, after prescribing antibiotics over the phone)
    - Who follows up the pathology results? (results faxed/sent directly to you, or rely on nurses at NH; same pathology service used for all patients?)

2. ANTIMICROBIAL USE AND PRESCRIBING BEHAVIOUR

- How do you (the GP) go about deciding on which antibiotic(s) to be prescribed and duration of treatment?
  - eg. antibiotic therapeutic guidelines
  - Do you usually specify a cessation date? (eg. further review of the patient, further blood tests, repeat urine, guidelines, number of tablets in the packet)
- What external help can you access for decision making?
  - eg. pharmacist, microbiologist, Infectious Disease registrar or consultant at a public hospital or private ID physician, other specialists
- Do you and your practice have access to ongoing education regarding infectious disease issues; if so, what type of educational support eg. newsletters, in-services by ID

physicians, reading articles, through representative from pharmaceutical companies, attendance at conferences, from Medicare Local/NPSMedicine Wise?

3. PERCEPTIONS TOWARDS ANTIMICROBIAL STEWARDSHIP INITIATIVES

- Understanding/knowledge: As you know, our project is about antimicrobial stewardship (AMS) in aged care facilities. What do you understand about AMS? (will define if unclear)

    - What are your thoughts on antibiotic prescribing in aged care facilities? (eg. it is a problem/not a problem, overuse/underuse, too much inappropriate use/appropriate most times) (eg. overuse/underuse, too much inappropriate use, lack of guidelines)

    - What are your (the GP) thoughts on antibiotic resistance organisims in aged care (eg. is it a growing problem/not a problem)?

    - What role do you think the aged care sector plays in generating resistant organisms? Do you think that antibiotic prescribing in aged care facilities contributes to this?

- Attitude towards a framework:

    - What are the key components needed, and barriers to optimising appropriate prescribing of antibiotics in aged care?
        - eg. better defined algorithms for nursing staff to use when a patient's condition changes
        - antibiotic therapeutic guidelines
        - additional ID specialist support/consultation automatically if an MDR organisms is identified
        - oversight about antibiotics (eg. in hospitals, teams have to justify use of certain antibiotics like ciprofloxacin, and sometimes cases are reviewed by an ID physician; do you think something like this is useful or feasible?)
        - education to nursing staff or families

    - Do you think GPs in general would support an AMS program in aged care facilities?

4. ANY OTHER QUESTIONS

- Are there any other issues that you feel we haven't talked about that you would like to mention?
