# Supplementary material for: Antimicrobial stewardship in residential aged care facilities: need and readiness assessment
Source: BMC Infect Dis. 2014 Jul 23;14:410. doi: 10.1186/1471-2334-14-410 (PMC4117949; doi:10.1186/1471-2334-14-410)
Supplement: Supplementary file 2 — Additional file 2: Semi-structured interview guide for nursing staff.(PDF 209 KB) [file 12879_2014_3703_MOESM2_ESM.pdf]

INTERVIEW GUIDE - NURSING STAFF

1. PRESCRIBING WORKFLOW/ ORGANISATIONAL CULTURE

- Questions in relation to the standard procedure and workflow involved in management of residents with an infection:

  - Under what condition you or other nurses may ask the doctor to start an antibiotic?
    Follow-up: UTI (clinical features make you suspect UTI, protocol to take full ward test- bimonthly/monthly)
    Chest infection (what s/s make you think it is chest infection?)

  - Were there situations doctor will order antibiotic over the phone? How frequent (% out of 100)? If so, when would the doctor come to review the particular resident?
    Follow-up: If weekend or night time, how's management? Do you see difference in infection management between locum doctors with usual GPs? Do GPs come for routine visit?

  - What are the guides for selection of antibiotics, say for UTI?
    Follow-up: institutional formulary, access to guidelines such as Antibiotic Therapeutic Guideline or other references in RACF, list of antibiotics to be used?

  - What are the situations where you see doctors seeking advices from external sources, and from where?

  - How do you keep tract of antimicrobial use/consumption patterns in your facility?
    Follow-up antimicrobial surveillance, routine audit/accreditation, etc (if there is routine surveillance, how effective is that to reduce/ control antimicrobial use?),

  - How many pathology (on average) your facility liaise with?
    Follow-up: retrieval of pathology report, protocol in-place for taking of urine culture (all taking urine cultures before antibiotic given?), documentation of pathology results, would doctors review C&S and change treatment?

  - How many pharmacies supplying the prescriptions? How often would the pharmacists intervene or recommend changes on the prescription of antibiotics?
    Follow-up: role of pharmacist in clinical decision making, medication review service by pharmacist (how frequent, do you see an increase of pharmacy medication review will be helpful?)

  - What aspects of current antimicrobial prescribing workflow/system which you think may need further improvement in your facility? Or What are the barriers/ difficulties you'd like to suggest for improvement?
    Follow-up: challenge faced and suggestions- communication with GPs or locum doctors? external support (ID, microbiology &pharmacy), availability of diagnostic facilities, knowledge of staff

2. ANTIMICROBIAL USE AND PRESCRIBING BEHAVIOUR

- Perceptions about current antibiotic use and prescribing behaviour:

  - Were there situations that you did not agree with the doctor's decision to prescribe antibiotics?
  - Can you think of an example when a resident wanted antibiotics but you felt unnecessary? Were there pressures from family members?

  - How long is the duration (on average) one course of antibiotics you commonly see? eg for UTI
  - Would you commonly see the doctor delay the antibiotic treatment for residents with cold/flu?
  - Was it frequent to see doctors delay or refuse treating residents with positive full ward test?
  - What group of residents would usually be prescribed long-term prophylactic antibiotics?
  - Is IV antibiotic commonly given to your residents?

- Do you see overuse or misuse of antibiotics is an issue in your NH? Or do you see a trend of increasing use of antibiotics in your facility?

- Is antimicrobial-resistance an issue in your NH? If yes, what might be the contributing factors?
  Follow-up: Was that often for you to come across cases where antibiotics have not been effective in treating residents with bacterial infection? Antibiotic resistance is a problem for the NH setting as a whole? Prompt if sufficient infection control strategies (hand hygiene, contact precaution, etc) taking place

- Any previous or existing efforts to improve the antibiotic prescribing at your facility?
- Are there room for improvement of any aspect of antibiotic use?

3. PERCEPTIONS TOWARDS ANTIMICROBIAL STEWARDSHIP INITIATIVES

- Perceptions towards AMS strategies in the RACF setting:

  - What does antimicrobial-stewardship (AMS) mean to you? (will define if unclear)
  - Should AMS be implemented in the RACF setting? If yes/no, why?

  - What intervention would you suggest to incorporate in an AMS initiative? Or what "shape" do you think the program should take?
    Follow-up: Education (whom to target)? Introduce clinical guidelines/protocol? Routine reviews of antibiotic use?

  - What would you imagine might be the major barriers in implementing AMS? How would you suggest to overcome?
    Follow-up: dedicated personnel (time/knowledge), Would staff be supportive? Would GPs taking advices?

  - How can AMS be made sustainable in this setting?
    Follow-up: what are facilitator/enablers for AMS implementation? External support vs. existing staff?

4. ANY OTHER QUESTIONS

- Are there any other issues that you feel we haven't talked about that you would like to mention?
