# Supplementary material for: Antimicrobial stewardship in residential aged care facilities: need and readiness assessment
Source: BMC Infect Dis. 2014 Jul 23;14:410. doi: 10.1186/1471-2334-14-410 (PMC4117949; doi:10.1186/1471-2334-14-410)
Supplement: Supplementary file 3 — Additional file 3: Semi-structured interview guide for pharmacists.(PDF 155 KB) [file 12879_2014_3703_MOESM3_ESM.pdf]

INTERVIEW GUIDE - PHARMACIST

1. PRESCRIBING WORKFLOW/ ORGANISATIONAL CULTURE

- On average, how much time in a week would you spend reviewing drug chart in the RACF? How many RACF do you attend to?
- In one week, how frequent were the orders of antibiotic placed (IV, IM vs. oral antibiotics) from a RACF on average?
- How would you decide if a prescription of antibiotic safe/appropriate to be dispensed? (explore if they would seek additional clinical information e.g. drug interaction, previous treatment with antibiotics, allergy, etc.)
- Were there sufficient clinical information provided during the order of antibiotic? If yes, what types of information were provided? If no, what type of info was missing?
- What were the common errors or drug related problems you spotted in the prescription of antibiotics?
- Do you commonly communicate with the doctors or nurses if you are unsure about the appropriateness of an antibiotic prescription?
- Is there a system to retrieve antibiotic orders for a particular RACF?
- Do you provide feedback about antibiotic use to the RACF? Do you have routine meeting with the NUM or facility managers regarding medicine supply? If yes, how frequent? Do you discuss/feedback antibiotic use?
- Do you involve in thorough medication review of RACF residents? If yes, how well the doctors will take the suggestion from pharmacist?
- Do you provide after-hour services (supply antibiotic, consultation, etc)?
- Were there challenges/difficulties in current system for you to communicate or recommend changes on the antibiotic prescriptions?

2. ANTIMICROBIAL USE AND PRESCRIBING BEHAVIOUR

- What might be the differences in antibiotic prescribing patterns between RACF residents vs. elderly community dwellers? (explore if higher use, broad spectrum antibiotic etc)
- How long (on average) is one course of antibiotic treatment? Have you ever receive prescriptions indicating two or more courses of antibiotic at the start?
- How common antibiotics were prescribed for prophylaxis purpose in this setting?
- Were there any changing prescribing patterns (types and number of antibiotics ordered) being observed for the last few years in RACF setting?
- What are your thoughts about antibiotic prescribing in the RACF? (eg overuse/underuse, lack of guidelines)
- Any aspect of antibiotic use which you sometime might query about its appropriateness or you don’t think that’s necessary?

3. PERCEPTIONS TOWARDS ANTIMICROBIAL STEWARDSHIP INITIATIVES

- What does antimicrobial stewardship (AMS) mean to you? (will define if unclear)
- If we were trying to set up an AMS program, how would you see that program working? Or what shape do you think the program should take? (education, clinical guideline/protocol, more frequent review/surveillance, etc)
- How do you see the role of pharmacist in facilitating the AMS program?
- What would you imagine might be the major barriers in implementing AMS? What solutions do you suggest? (explore the facilitator/ enables for AMS)

4. ANY OTHER QUESTIONS

- Are there any other issues that you feel we haven’t talked about that you would like to mention?
